# LuxCDE-luxAB-based promoter reporter system to monitor the *Yersinia enterocolitica* O:3 gene expression *in vivo*

**DOI:** 10.1371/journal.pone.0172877

**Published:** 2017-02-24

**Authors:** Elif Bozcal, Melih Dagdeviren, Atac Uzel, Mikael Skurnik

**Affiliations:** 1 Istanbul University, Faculty of Science, Department of Biology, Basic and Industrial Microbiology Section, Istanbul, Turkey; 2 Ege University, Faculty of Science, Department of Biology, Basic and Industrial Microbiology Section, Izmir, Turkey; 3 Department of Bacteriology and Immunology, Medicum, and Research Programs Unit, Immunobiology, University of Helsinki, Helsinki, Finland; 4 Ege University, Faculty of Science, Department of Biology, General Biology Section, Izmir, Turkey; 5 Ege University, Center for Drug Research and Development and Pharmacokinetic Applications, Izmir, Turkey; 6 Division of Clinical Microbiology, Helsinki University Hospital, HUSLAB, Helsinki, Finland; Beijing Institute of Microbiology and Epidemiology, CHINA

## Abstract

It is crucial to understand the *in vitro* and *in vivo* regulation of the virulence factor genes of bacterial pathogens. In this study, we describe the construction of a versatile reporter system for *Yersinia enterocolitica* serotype O:3 (YeO3) based on the *luxCDABE* operon. In strain YeO3-*luxCDE* we integrated the luciferase substrate biosynthetic genes, *luxCDE*, into the genome of the bacterium so that the substrate is constitutively produced. The *luxAB* genes that encode the luciferase enzyme were cloned into a suicide vector to allow cloning of any promoter-containing fragment upstream the genes. When the obtained suicide-construct is mobilized into YeO3-*luxCDE* bacteria, it integrates into the recipient genome via homologous recombination between the cloned promoter fragment and the genomic promoter sequence and thereby generates a single-copy and stable promoter reporter. Lipopolysaccharide (LPS) O-antigen (O-ag) and outer core hexasaccharide (OC) of YeO3 are virulence factors necessary to colonization of the intestine and establishment of infection. To monitor the activities of the OC and O-ag gene cluster promoters we constructed the reporter strains YeO3-P_oc_::*luxAB* and YeO3-P_op1_::*luxAB*, respectively. *In vitro*, at 37°C both promoter activities were highest during logarithmic growth and decreased when the bacteria entered stationary growth phase. At 22°C the OC gene cluster promoter activity increased during the late logarithmic phase. Both promoters were more active in late stationary phase. To monitor the promoter activities *in vivo*, mice were infected intragastrically and the reporter activities monitored by the IVIS technology. The mouse experiments revealed that both LPS promoters were well expressed *in vivo* and could be detected by IVIS, mainly from the intestinal region of orally infected mice.

## Introduction

The bacterial genus *Yersinia* has three species known to cause human infections: *Yersinia enterocolitica*, *Y*. *pseudotuberculosis* and *Y*. *pestis*. *Y*. *enterocolitica* has been divided into six biotypes, biotype 1A includes the non-pathogenic strains and biotypes 1B, 2, 3, 4, and 5 include the pathogenic strains. The biotype 1B to 5 strains usually carry a 70 kb *Yersinia* virulence plasmid (pYV) that encodes several well-characterized virulence determinants [1]. The chromosomally encoded virulence factors include Ail (attachment and invasion locus), invasin, *Yersinia* stable toxin and lipopolysaccharide (LPS) [2–4]. Most pathogenic *Y*. *enterocolitica* strains that cause human yersiniosis belong to bioserotypes 1B/O:8, 2/O:5,27, 2/O:9, 3/O:3, 4/O:3 [5]. Yersiniosis is generally a self-limiting food-borne enteric infection with diarrhea, and occurs mostly in young children. The infection may be followed by sequelae such as reactive arthritis or erythema nodosum [6]. *Y*. *enterocolitica* mainly colonizes the Peyer’s patches (PPs) in the small intestine (INT), the mesenteric lymph nodes (MLNs), and sometimes also disseminates to deeper tissues such as spleen (SP) and liver (LI) in mice [7, 8].

LPS, also known as endotoxin, is an important bacterial surface antigen used by the host immune system in recognition of the pathogen. In *Y*. *enterocolitica* O:3 (YeO3) different structural parts of LPS are virulence factors and play a role immediately after the entry to the host [9, 10]. LPS is a large molecule consisting of a lipid A (LA) covalently linked to a polysaccharide composed in inner core (IC), outer core (OC) and O-antigen (O-ag) or O-polysaccharide (OPS). YeO3 does not produce the conventional Gram-negative LA-IC-OC-O-ag type LPS molecules but has two types of LPS molecules; LA-IC substituted either with O-ag or with OC but not by both, i.e., LA-IC-O-ag or LA-IC-OC [11, 12]. In addition to the biological activity of LA as endotoxin, in some bacteria O-ag is also significant for colonization of host tissues, resistance to complement mediated killing and for initial interactions between the bacterium and the host. The rough, *i*.*e*., O-ag–lacking, strains of *Y*. *enterocolitica* serotypes O:3 and O:8 are 50–100 times less virulent than wild type strain in orally infected mice [13, 14]. OC of YeO3 is required for full virulence and strongly suggested that it provides resistance against host defense mechanisms [10].

Several virulence factors of *Yersinia* species are temperature-regulated; however, the regulation mechanisms are poorly understood and need to be studied further. For instance, the stable enterotoxin of *Yersinia* (Yst) is optimally produced at 22–25°C, however, the surface adhesins *e*.*g*., Ail and YadA are produced at 37°C [15]. Moreover, *in vitro*, the production of LPS OC and O-ag is temperature-regulated, *i*.*e*., bacteria grown at 22°C produce more OC and O-ag than bacteria grown at 37°C [9, 12]. Furthermore, in *Y*. *enterocolitica* serotype O:8 absence of O-ag affected the expression of other virulence factors [16]. On the other hand, in YeO3, abundant O-ag production blocked the Ail protein and thereby affected the complement resistance of the bacteria underlining the importance of proper regulation of the LPS components to functionality of other virulence factors [17, 18]. Finally, it has been speculated that during infection the O-ag expression might be repressed after the colonization to deeper tissues to allow proper exposure of other surface-located virulence factors [19].

Bioluminescence imaging makes it possible to follow the progression of the infection in animal models [20] for example after a reporter gene has been cloned under the control of a bacterial promoter. As bacteria mostly respond to changes through transcriptional regulation virulence gene promoter reporters can be used to monitor the infection process *in vivo* to elucidate how bacteria respond to environmental changes [21]. The *in vivo* imaging system (IVIS) is an efficient approach to study bacterial infections at real time in live animals [20, 22]. IVIS can be used with various reporter systems including the green fluorescent protein (GFP) and bacterial luciferase encoded by the *luxAB* genes. While the GFP produces fluorescence upon UV-light illumination, the luciferase requires the (exogenous) aldehyde substrate for the light emission [23]. This requirement is overcome by the employment of the whole *luxCDABE* operon where the *luxCDE* genes encode the enzymes of the fatty acid reductase complex to generate the tetradecanal substrate for the bioluminescence reaction [24, 25]. Both GFP and bacterial *luxAB* reporters have been used to study *Yersinia* infections in mice [25].

In light of the above information, to understand the regulation of the YeO3 O-ag and OC gene clusters *in vitro* and *in vivo* we constructed a versatile promoter reporter system for YeO3. The YeO3 reporter system was constructed by integrating the *luxCDE* genes into the genome of the bacterium to constitutively supply the bacteria with the luciferase substrate. To avoid the potential hazards of the copy number effects of plasmid-carried promoter reporters, a promoterless *luxAB* suicide vector was constructed for promoter cloning. The latter allows the integration of the promoter reporter as a single-copy into the bacterial genome via homologous recombination. To demonstrate the utility of the system the LPS OC and O-ag gene cluster promoter reporters were constructed.

## Materials and methods

### Bacterial strains, plasmids and growth conditions

The bacterial strains and plasmids used in this work are listed in Table 1. Bacteria were grown aerobically in Lysogeny Broth (LB) [26] or on *Yersinia* selective agar (CIN) (Oxford, UK) at either 37°C or at room temperature (RT, ca. 22°C). LB plates were prepared by adding 1.5 g of Bacto agar (Difco) to 1 L of LB. Antibiotics were used when needed at the following concentrations: streptomycin (Str) 50 μg/ml, chloramphenicol (Clm) 100 μg/ml, ampicillin (Amp) 50 μg/ml.

**Table 1:** Bacterial strains and plasmids.

Bacterial strains and plasmids	Description	Reference
***Y*. *enterocolitica* strains**		
YeO3 (6471/76)	Serotype O:3, patient isolate, wild type, pYV^+^. (DSMZ accession number DSM 23248)	[27]
YeO3-LuxCDE-1	*Y11_01521-luxCDE* transcriptional fusion	This work
YeO3-LuxCDE-2	*Y11_01531-luxCDE* transcriptional fusion	This work
YeO3-LuxCDE-1::pEfB2	YeO3-luxPoc, *Poc*::*luxAB*, OC gene cluster promoter reporter	This work
YeO3-LuxCDE-1::pEfB3	YeO3-luxPop1, *Pop1*::*luxAB*, O-ag gene cluster promoter reporter	This work
YeO3-LuxCDE-2::pEfB2	*Poc*::*luxAB*, OC gene cluster promoter reporter	This work
***E*. *coli* strains**		
S17-1λpir	*thi pro hsdR*^-^ *hsdM*^+^ *recA*::RP4-2-Tc::Mu-Km::Tn7, (λ*pir*), Str^R^	[28]
SY327 λpir	Δ(*lac pro*) *argE* (Am) *rif nalA recA56* (λ*pir*)	[29]
SM10λpir	*thi thr leuB tonA lacY supE recA*::RP4-2-Tc::Mu-Km *(*λ*pir)*	[30]
ω7249	β2163Δnic35 Km^R^ Ery^R^	[31]
DH10B	F^-^ *mcrA Δ(mrr-hsd* RMS-*mcrBC*), ϕ80*lacZ*ΔM15 Δ*lac*X74, *deoR*, *recA1 endA1 araΔ139Δ(ara*, *leu)7697 galU*, *galK λ*^-^ *rpsL nupG λ*^-^ *tonA*	Life Technologies
**Plasmids**		
pTetlux1	Source of *luxCDABE*	[32]
pSW23T	Suicide plasmid, *oriR6K*, *oriT*, Clm^R^	[33]
pKNG101	Suicide plasmid, *oriR6K*, *oriT*, *sacB*, Str^R^,	[34]
pEfB1	pSW23T derivative with promoterless *luxAB* genes	This work
pEfB2	pEfB1 derivative with *Poc* cloned upstream of *luxAB*	This work
pEfB3	pEfB1 derivative with *Pop1* cloned upstream of *luxAB*	This work
pTetLuxCDE	pTetLux1-Δ*luxAB*	This work
pSW23T-ΔM	pSW23T derivative with the MfeI site knocked out	This work
pEfB4	*Y11_01521-Y11_01531* fragment cloned into pSW23T-ΔM	This work
pEfB5	pEfB4 derivative with a *MfeI* site introduced between the *Y11_01521* and *Y11_01531* genes	This work
pEfB6-a	*luxCDE* cassette cloned into *MfeI* site of pEfB5 fused to *Y11_01521*	This work
pEfB6-b	*luxCDE* cassette cloned into *MfeI* site of pEfB5 fused to *Y11_01531*	This work
pEfB7-a	*Y11_01521-luxCDE -Y11_01531* fragment cloned into pKNG101	This work
pEfB7-b	*Y11_01531-luxCDE -Y11_01521* fragment cloned into pKNG101	This work

### Construction of genomic luxCDE integration vectors

We first removed the MfeI restriction site from pSW23T (Table 1) by blunting the 5’-overhangs of the MfeI-digested plasmid with T4 DNA polymerase followed by self-ligation and the obtained suicide vector was named pSW23T-ΔM. The *Y11_01511 –Y11_01531* region of YeO3 genome was amplified by PCR using primers 1521-F1M and 1521-R1 (Table 2) to generate a 1485 bp fragment with BamHI restriction sites. This fragment was digested with BamHI and ligated with BamHI digested and phosphatase-treated pSW23T-ΔM. The ligation mixture was electroporated into *E*. *coli* Sy327λpir and obtained Clm^R^ transformants were screened by colony PCR using the primers 1521-F1M and 1521-R1. Plasmid DNA was isolated from a few PCR-positive clones and digested with BamHI to confirm the insert. The obtained 3,302 bp plasmid was named pEfB4. To introduce an MfeI site (compatible with EcoRI) in the middle of the inserted fragment between the *Y11_01511 `–Y11_01531* genes, pEfB4 was amplified by plasmid PCR with MfeI site carrying primers 1521-F3 and 1521-R3. The obtained 3.3 kb PCR fragment was digested with MfeI, self-ligated, transformed into *E*. *coli* Sy327λpir and the resulting plasmid was named pEfB5.

**Table 2:** Primers used in this study.

Primer	Sequence (restriction sites in bold)	Description/target	Size of PCR product (bp)
Lux2-F	cgc**ggatcc**ttcaagctgctgctttgttg	*luxAB* cassette	2081
Lux2-R	cgc**gaattc**actcaaa tagcaatataaggactct		
O3ocP-fS	cgg**gtcgac**atgagggcccatagaccttt	OC gene cluster promoter (*Poc*)	445
O3ocP-rE	cggc**gaattc**tcccttgtaacaaaagcaaaga		
O3op1-fS	cgg**gtcgac**tctttaagcatggcggtcagt	O-ag gene cluster promoter (*Pop1*)	409
O3op1-rE	cggc**gaattc**gccccaaaaatgagaatgaa		
Lux1-F	agtgaacgaatccccaggag	Deletion of *luxAB* from pTetlux1	8139
Lux1-R	ttaagacagagaaattgcttgattttca		
1521-F1M	ggc**ggatcc**ggtgaaaggcgatgatgaaccc	*Y11_01511-Y11_01531* fragment	1485
1521-R1	ggc**ggatcc**tgccaatcagcatcactgt		
1521-F3	ggc**caattg**ttttacttaatagccctcaactcagacag	Introduction of MfeI site to pEfB4	3308
1521-R3	ggc**caattg**tatttagtgcggtaattgggcttgttc		
LuxC-F	gggcaaagatggaaaaatga	*luxC*	498
LuxC-R	tccccaagcgacaataacat		
ms-09	cctatccctcttctatgggag	*yadA*	494
ms-10	gctactccatctttctgggcg		

Plasmid pTetLux1 carrying the *luxCDABE* operon was used as a template in plasmid PCR using primers Lux1F and Lux1R. The PCR linearizes the plasmid without the *luxAB* genes. The fragment was self-ligated to close the gap between *luxD* and *luxE* and the generated plasmid was named pTetLuxCDE. The *luxCDE* cassette was carried in it within a 4.8 kb EcoRI fragment. The latter was released from pTetLuxCDE with EcoRI digestion and ligated with MfeI-digested pEfB5. The ligation mixture was transformed into *E*. *coli* Sy327λpir and Clm^R^ transformants were screened by PCR using *luxC*-specific primers LuxC-F and LuxC-R (Table 2). Plasmids from *luxC*-positive clones were isolated, analysed by restriction digestions, and two of the obtained plasmids were called pEfB6-a and pEfB6-b as in them the *luxCDE* cassette had been ligated in opposite orientations between the *Y11_01521* and *Y11_1531* genes.

Finally, the *Y11_01521-luxCDE-Y11_01531* fragments of pEfB6-a and pEfB6-b were cloned as BamHI-fragments into suicide vector pKNG101 (Table 1) that carries the *sacB* gene to facilitate the positive selection of allelic exchange. The ligation mixtures were transformed into Sy327λpir, Str^R^ transformants were screened by colony PCR, the insertion of the correct fragment was confirmed by digestion of the isolated plasmids with BamHI, and the obtained plasmids were named pEfB7-a and pEfB7-b.

#### Construction of promoter-luxAB integration vectors

The *luxAB* genes were amplified by PCR with primers Lux2-F and Lux2-R (Table 2) that include BamHI and EcoRI restriction sites, respectively, using plasmid pTetlux1 as template. The amplified *luxAB* fragment was digested with EcoRI and BamHI and ligated with similarly digested suicide vector pSW23T. The ligation mixture was transformed into *E*. *coli* S17λpir and Clm^R^ transformants were selected. Plasmids from the transformants carrying the *luxAB* fragment in correct orientation were identified by colony PCR and digestion with BamHI and EcoRI, and one of the obtained plasmids was named as pEfB1. PCR was used to amplify ca. 400 bp fragments carrying the promoter regions of the YeO3 OC and O-ag gene clusters (designated as *Poc* and *Pop1*, respectively). These promoters are located upstream of genes *Y11_ 19991* and *Y11_16781* of strain Y11, respectively (accession number: FR729477). The primer pairs were O3ocP-fS and O3ocP-rE, and O3op1-fS and O3op1-rE (Table 2) for *Poc* and *Pop1*, respectively, and YeO3 genomic DNA was used as template. The primers introduced SalI and EcoRI restriction sites up- and down-stream of the promoters, respectively. The SalI and EcoRI digested promoter fragments were ligated with similarly digested pEfB1 and transformed into *E*. *coli* S17λpir. Clm^R^ transformants were selected. Plasmids from the transformants carrying the promoter fragments in the correct orientation were identified using PCR and restriction digestions with NsiI. The resulting *Poc* and *Pop1* promoter-fragment carrying plasmids were named as pEfB2 and pEfB3, respectively.

#### Mobilization of suicide vectors by conjugation and screening for mutant strains

The suicide constructs pEfB7-a and pEfB7-b were mobilized from *E*.*coli* SM10λpir (Str^R^) to YeO3 wild type strain 6471/76 by conjugation. The donor bacteria were grown aerated overnight at 37°C in 5 ml of LB supplemented with Str. The YeO3 bacteria were grown with shaking overnight in 5 ml of LB at RT. The donor and recipient cultures were centrifuged (1500 ×*g*, 15 min), the pellets washed with phosphate-buffered saline (PBS, pH 7.2), and resuspended into PBS to an OD_600_ ~1. Mating mixtures containing 50 μl aliquots of the donor and recipient suspensions were mixed (2:1) on LB plates. After 24 h mating at RT, the bacteria were collected into 1 ml of PBS. To select merodiploid YeO3 transconjugants in which the suicide construct had integrated via homologous recombination into the bacterial genome, aliquots of the suspensions and dilutions thereof were spread on CIN-Str plates. Bacteria from 10 individual Str^R^ colonies from both matings (*i*.*e*., of pEFB7-a or pEFB7-b with YeO3) were pooled and grown in LB without any antibiotics for three passages at 25°C to complete the allelic exchange via a second site recombination. The culture from the last passage was 1:5 diluted with fresh LB supplemented with 5% sucrose and incubated at RT for 3 h. The recombinants that had lost the *sacB* gene along with the suicide vector would survive the incubation with sucrose were screened for Str^S^ *luxC*-positive clones. From both matings a representative clone was selected and named YeO3-LuxCDE-1 and YeO3-LuxCDE-2 carrying the *luxCDE* genes transcriptionally fused to the *Y11_1521* and *Y11_1531* genes, respectively. In both, the exchange of the *Y11_01521-Y11_01531* fragment to the *Y11_01521-luxCDE-Y11_01531* fragment was confirmed by PCR using primers 1521-F1M and 1521-R1 (Table 2).

#### Construction of the promoter reporter strains

The *Poc* and *Pop1* promoter suicide vectors pEfB2 and pEfB3 were mobilized into YeO3-LuxCDE-1 and YeO3-LuxCDE-2 by conjugation as described above. Clm^R^ merodiploid transconjugants were selected carrying in the bacterial genome the *luxAB* cassette integrated by homologous recombination under the OC and O-ag gene cluster promoters. PCR using primers Lux2-F and Lux2-R (Table 2) was used to check that the suicide vector had integrated into the recipient strains. While we failed to isolate pEfB3 transconjugants in YeO3-LuxCDE-2, the three other reporter strains YeO3-LuxCDE-1::pEfB2 (called hereafter YeO3-P_oc_::*luxAB*), YeO3-LuxCDE-1::pEfB3 (called hereafter) YeO3-P_op1_::*luxAB*, and YeO3-LuxCDE-2::pEfB2 were isolated (Table 1). Light production in both YeO3-LuxCDE-1/pEfB2 and YeO3-LuxCDE-2/pEfB2 was identical, therefore only the former strain was used in further studies.

#### Bioluminescence and growth phase

The reporter strains YeO3-P_*oc*_::*luxAB* and YeO3-P_op1_::*luxAB* were inoculated in LB medium supplemented with Clm and grown for 15 hr at RT and 37°C. Bacteria were diluted to OD_600_ of 0.2 in a total volume of 50 ml of fresh medium pre-incubated to respective temperature, and grown aerated for 24 hr at RT and 37°C. Culture samples were harvested in triplicate at 0, 2, 4, 6, 8, 10, and 24 hr time points to determine OD_600_ and measure bioluminescence (as relative light units, RLU). The bioluminescence values were measured using Chameleon plate reader (HIDEX, Turku, Finland). Experiments were performed with at least 3 biologial and 3 technical replicates.

#### Sensitivity of the lux reporter detection

Overnight cultures of the reporters were diluted 1:100 (OD_600_ ~0.03) in LB-Clm and grown at RT and 37°C until OD_600_ reached 0.2 (~10^8^ cfu/ ml). The cultures were serially diluted in PBS (pH 7.45) down to ~10^2^ cfu/ml and 0.2 ml aliquots were transferred in triplicate to a 96-well plate. The bioluminescence at both temperatures was measured using the Chameleon plate reader (HIDEX). The corresponding bacterial counts were determined by plating serial dilutions on LA-Clm plates. The RLU from wells with 0.2 ml PBS without bacteria was used as the background value that was subtracted from the RLU values produced by the bacteria.

#### Growth curves

The growth curves of the bacterial strains were determined using the Bioscreen C incubator (Growth Curves Ab Ltd). Overnight (ca. 16 hr) cultures of the bacteria were diluted with fresh LB medium to an OD_600_ of 0.2. Aliquots of 200 μl were distributed into honeycomb plate wells and grown for 15 hr at RT and 37°C with continuous shaking. The OD_600_ of the wells was measured every 15 min. The averages were calculated from values obtained for the bacteria grown in 6 parallel wells. Experiments were performed with at least 3 experimental and 6 technical replicates.

#### Immunoblotting

Immunoblotting was performed as described earlier [17] with minor modifications. Briefly, bacteria were grown overnight in 5 ml LB at RT and 37°C by shaking at 200 rpm. The OD_600_ of the culture was adjusted to ~1 with LB, bacteria from a 500 μl aliquot were centrifuged, resuspended into 100 μl SDS sample lysis buffer and heated at 100°C for 10 minutes. After cooling the samples on ice, 10 μl aliquots were separated in gel electrophoresis using the Hoefer miniVE Vertical Electrophoresis System (Amersham, Biosciences) with 12% resolving and 5% stacking polyacrylamide gels. The separated samples were transferred to nitrocellulose membranes by semi-dry transfer apparatus (Blot module; Amersham Biosciences) using the recommended transfer buffer. The membrane was blocked with blocking buffer including 5% fat-free milk. The membranes were then incubated overnight at 4°C with mouse monoclonal antibodies (mAb) specific for O-ag (Tom A6) and OC (2B5) [35], diluted 1:25 and 1:10, respectively. The horse radish peroxidase (HRP)-conjugated goat anti-mouse immunoglobulin antibodies (P0447, dilution 1:2000, DAKO) were used as secondary antibodies. The bound HRP was detected using the enhanced chemiluminescence (ECL) developing solution applied onto the membrane followed by exposure of an X-ray film. This experiment was performed with 2 biological replicates.

#### The IVIS experiment

Mouse experiments at University of Helsinki were under the ethical permission granted by the Animal Experiment Board in Finland (Decision No:ESAVI/5893/04.10.03/2012), and at Ege University under the permit (No: 2015–004) from the Animal Experiment and Ethics Committee of Ege University. Female BALB/c mice (6–8 weeks old, 18.65±0.20 g), were purchased from Kobay A.S. (Ankara-Turkey), and were kept in quarantine for one week after arrival from the supplier. Handling of each mouse during the experiments was carried out according to the European Communities Council directives (86/609/EEC). The mice were kept under standard colonial conditions (4/5 mice per cage, 21±1°C, 12 hr day/night cycle), standard mouse pellet (except saline/desferal injection day) and tap water were available *ad lib*. One day before the bacterial infections, 10 mg of the iron chelator desferrioxamine (Desferal, Novartis^®^) dissolved in 100 μl sterile dH_2_O was injected intraperitoneally (i.p.) into half of the mice; the remaining mice formed the saline-treated (SLN) group. Desferal (DSF) enhances the virulence of *Y*. *enterocolitica* [10, 36]. The bacteria for the infection experiments were prepared as described earlier [10]. The presence of the *Yersinia* virulence plasmid (pYV) in each strain was verified by the autoagglutination of the bacteria when grown at 37°C in MedECa medium as described [17, 37]. The actual bacterial concentrations in each experiment were determined by plating serial dilutions of the inocula. The mice were kept without solid food for 12 h before bacterial challenge [10]. Saline was injected to control mice to eliminate the difference of injection stress.

The mice were inoculated i.g. via gavage with 100 μl of the bacterial suspension in PBS (pH 7.45). The inocula were 1.1×10^9^ CFU and 2×10^9^ CFU for YeO3-P_oc_::*luxAB* and YeO3-P_op1_::*luxAB*, respectively. For the imaging, the mice were anesthetized with ketamine (50 mg/kg)–xylazine (10 mg/kg) and images were captured on day 1 and 5 post-infection using the IVIS Spectrum device (Caliper Life Sciences, Hopkinton-MA). The best imaging results were achieved using high resolution and a 5 min exposure time. The results, reported as radiance (p/sec/cm^2^/sr), were acquired by a computer using the Living Image Software (PerkinElmer, USA). The images were stored and analyzed with the connected computer. On day 5 post-infection, after capturing the images by IVIS, the mice were sacrificed by cervical dislocation while still under anesthesia. Piece of LI, the INT, the MLNs and the SP of each animal were placed on a Petri dish and images were captured with IVIS using a 1 min exposure time. The PPs were then prepared from the small intestines, and all the tissues were weighted and stored frozen in 20% glycerol at -20°C overnight. Identical tissues of each mouse group were combined and homogenized in ice cold PBS (3 ml/g of tissue) with Potter-Elvehjem homogenizer on ice. 500 μl aliquots of the homogenate were recovered from each group for bacterial counts. Two 20 μl aliquots of serially diluted homogenates were plated immediately on CIN-Clm plates to determine the bacterial numbers in the homogenates by the drop test described earlier [38, 39].

### Statistics

GraphPad Prism 5.0 (SanDiego, CA, USA) program was used for statistical analysis. One way ANOVA and Student’s t-tests were performed and post-hoc tests were applied when needed. P- values *≤*0.05 were considered significant.

## Results

### The design of the reporter strains

**Fig 1:**
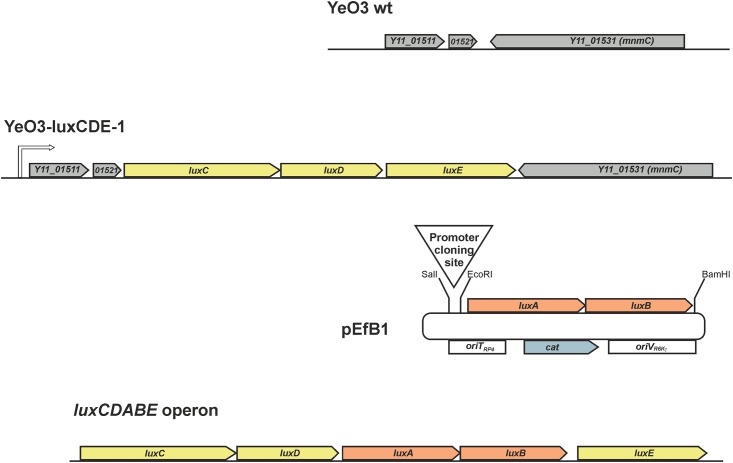
Construction of the luciferase reporter system for YeO3. The promoterless *luxCDE* genes were introduced between the *Y11_01521* and *Y11_01531 (nmnC)* genes of the wild type (WT) strain to obtain the strain YeO3-LuxCDE-1 with the *luxCDE* genes transcriptionally fused to *Y11_01521*. In strain YeO3-LuxCDE-2 (not shown) the *luxCDE* genes are in opposite direction and transcriptionally fused to *Y11_01531*. The LuxC, LuxD and LuxE proteins are the transferase, synthetase, and reductase to produce from fatty acids the long-chain aldehyde substrate for the LuxAB luciferase. The *luxAB* genes were cloned into the suicide vector pSW23T between the EcoRI and BamHI sites to obtain pEfB1. Cloning of any YeO3 promoter fragment into the pEfB1 promoter cloning site produces a promoter-*luxAB* suicide construct. When the promoter suicide construct is mobilized into strain YeO3-LuxCDE-1, it will via homologous recombination generate a reporter strain where the promoter activity translates into LuxAB expression that can be detected as bioluminescence. The organization of the *Photorhabdus luminescens luxCDABE* operon is depicted at the bottom.

In this work, we engineered a bacterial *luxCDABE* operon-based promoter reporter system for YeO3 such way that the luciferase encoding *luxAB* genes and the substrate biosynthetic enzymes encoding *luxCDE* genes were separated. In addition, we wanted to incorporate both the *luxCDE* and *luxAB* genes into the bacterial genome to avoid possible copy-number effects associated in plasmid-borne reporter systems. To generate a versatile system able to generate reporters to any chosen promoter, a strain expressing the *luxCDE* genes from a non-relevant location in the genome was needed. To this end, we first identified from the YeO3 genome a constitutively transcribed locus where the *luxCDE* genes could be transcriptionally fused. For this we utilized the RNA-seq data of YeO3 grown both at 22°C and 37°C [40]. Based on the RNA-seq data the genes *Y11_01521* and *Y11_01531* (YeO3 strain Y11, accession number: FR729477) are both expressed constitutively and at moderate level. The gene *Y11_01531* encodes for a bifunctional tRNA-methyltransferase MnmC, and the gene *Y11_01521*, for a hypothetical protein. The genes appropriately face each other being the last genes in their operons (Fig 1). Thus, we reasoned that insertion of the *luxCDE* genes between the genes would generate a transcriptional fusion irrespective the *luxCDE* orientation. In this line, the strains YeO3-LuxCDE-1 and YeO3-LuxCDE-2 were constructed to produce constitutively the luciferase substrate biosynthetic enzymes (Fig 1). Upon introduction of the *luxAB* genes into these strains, light should be produced proportional to the promoter strength upstream the *luxAB* genes. As a tool to integrate the *luxAB* genes into the YeO3 genome we constructed a promoter cloning suicide vector pEfB1. Any promoter of YeO3 can be cloned to the cloning site upstream the promoterless *luxAB* genes in pEfB1 (Fig 1).

### Reporter strain fitness and sensitivity

**Fig 2:**
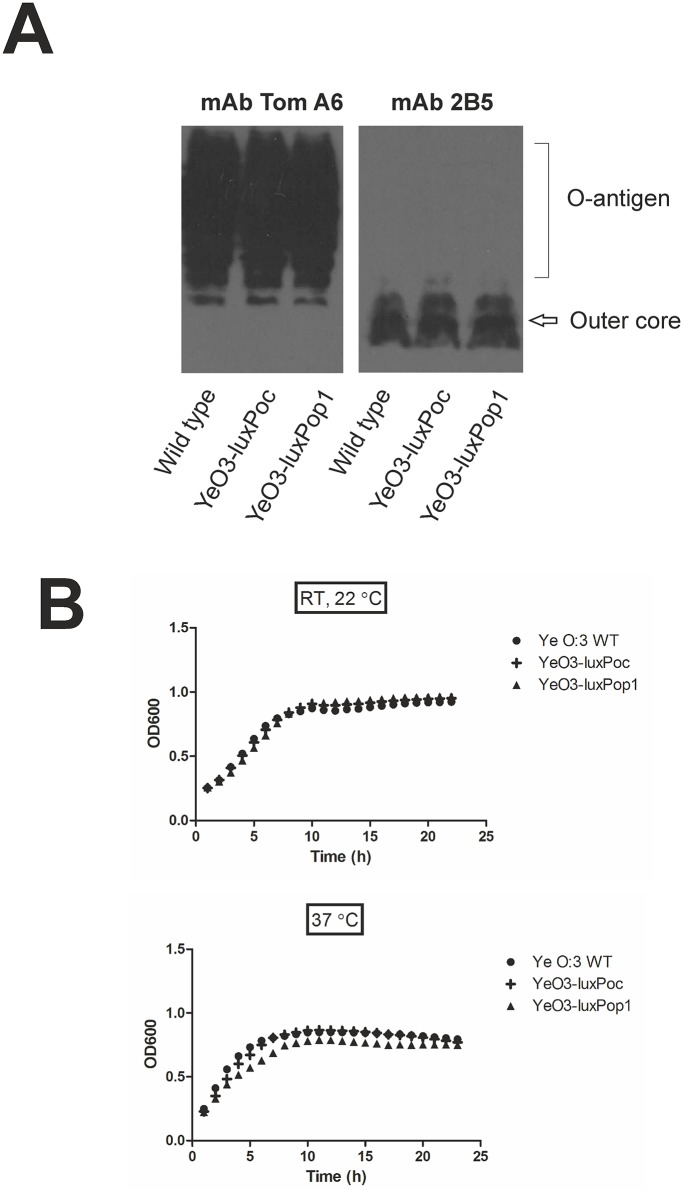
Characterization of the influence of promoter reporter constructions on the LPS expression and fitness of the *Y*. *enterocolitica* O:3 reporter strains. **(A).** Western blotting analysis of LPS expression profiles in wild type and reporter strain bacteria grown at 22°C. The O-antigen and OC specific mAbs Tom A6 and 2B5, respectively, were used as primary antibodies **(B).** Growth curves of wild type and promoter reporter bacteria in LB at 37°C and 22°C.

The YeO3-P_oc_::*luxAB* and YeO3-P_op1_::*luxAB* reporter strains (Table 1) were constructed to allow monitoring the LPS OC and O-ag gene cluster gene expression, respectively. To verify that the merodiploid situation with the promoter-pEfB1 plasmid integrated into the host genome did not disturb the expression of the target gene clusters, the O-ag and OC production in the YeO3-P_oc_::*luxAB* and YeO3-P_op1_::*luxAB* reporter strains was compared to that of the wild type strain by immunoblotting (Fig 2A). Both strains produced O-ag and OC identical to the wild type strain, furthermore, the growth curves of the reporter strains were identical to that of the wild type strain (Fig 2B) indicating that the reporter systems did not influence the fitness of the bacteria. To determine the minimal number of bacteria required to produce light above the background, light was measured from different numbers of reporter bacteria grown to logarithmic phase at 22°C and 37°C (Table 3). 10^2^ and 10^3^ CFU per well already produced RLU values slightly above the PBS background (~10 RLU), 10^4^ CFU produced RLU values clearly above the background.

**Table 3:** Light detection using the Chameleon plate reader from different numbers of *Y*. *enterocolitica* O:3 OC and O-ag gene cluster promoter reporter bacteria.

No of bacteria	YeO3-P_oc_::*luxAB* (RLU)				YeO3-P_op1_::*luxAB* (RLU)			
	22°C	SEM*	37°C	SEM*	22°C	SEM*	37°C	SEM*
10^7^	5317.66	±32.05	12453.66	±130.81	9870.33	±389.08	7838.66	±104.14
10^6^	533.66	±20.18	1328.33	±2.96	989.00	±6.50	801.00	±14.89
10^5^	95.33	±3.28	163.00	±1.52	161.66	±6.69	108.66	±4.81
10^4^	46.66	±1.85	31.33	±5.23	47.33	±0.33	31.66	±1.78
10^3^	16.66	±3.66	22.33	±2.40	34.66	±4.66	27.00	±4.54
10^2^	8.33	±2.84	14.33	±2.33	26.66	±3.17	10.00	±0.47
10^1^	8.66	±0.88	7.33	±2.18	13.00	±0.00	6.33	±1.36

*standard error of mean

**Fig 3:**
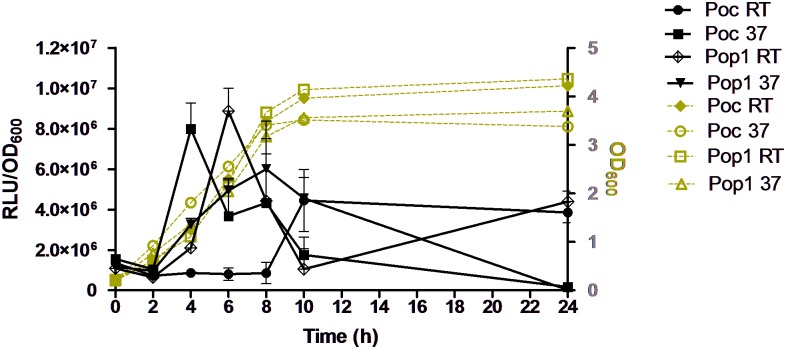
The *in vitro* OC and O-ag gene cluster expression profiles of the *Y*. *enterocolitica* O:3. reporter bacteria YeO3-P_oc_::*luxAB* (Poc) and YeO3-Pop1::*luxAB* (Pop1) during growth in 50 ml LB at 22°C and 37°C. The data points show averages of three replicates and the bars above the data points show the +SD values. RLU, relative light units.

Next we studied whether the growth phase and/or the temperature influence the Poc and Pop1 activities. To this end, bacterial cultures were adjusted to OD_600_ = ~0.2, incubated at 22°C and 37°C, and the RLU/OD_600_ values were determined at different time points during the incubation (Fig 3). At 37°C both promoters appeared to be most active during the exponential growth phase which would be logical as that is the phase where new LPS molecules are synthesized [9], however, the two promoters behaved differently at RT. In contrast to Pop1, the Poc reporter was not active at RT during log phase. When the bacteria grown at RT reached the early stationary phase at 8–10 hr, both promoter activities were relatively low, however, both were more active at 24 hr at RT than in early stationary phase (8–10 hr). On the other hand, at 37°C, both promoters were very active during the log phase but almost completely repressed at 24 hr.

### *In vivo* expression studies

The reporter strains YeO3-P_oc_::*luxAB* and YeO3-P_op1_::*luxAB* were used in mouse infection experiments. In a preliminary experiment carried out with a few mice it was established that the reporter bacteria produced detectable amount of light *in vivo* (data not shown). This allowed us to carry out IVIS experiments on experimentally infected mice. Altogether 37 mice were used in the *in vivo* imaging experiments. Traditionally, in LD_50_ experiments with YeO3, to enhance the pathogenicity of the bacteria, the mice are pre-treated with the iron-chelating compound DSF [10, 36]. To ascertain the detectability of the bacteria by IVIS, half of the mice were given DSF and the other half was pre-treated with 0.9% NaCl (SLN). Altogether four experimental groups were generated: OC-SLN (n = 8), OC-DSF (n = 9), OA-SLN (n = 10) and OA-DSF (n = 10). The groups infected with YeO3-Poc::*luxAB* were designated as OC-SLN and OC-DSF, and those with YeO3-Pop1::*luxAB*, as OA-SLN and OA-DSF. The *in vivo* reporter activities were monitored as light emission on days 1 and 5 post-infection.

**Fig 4:**
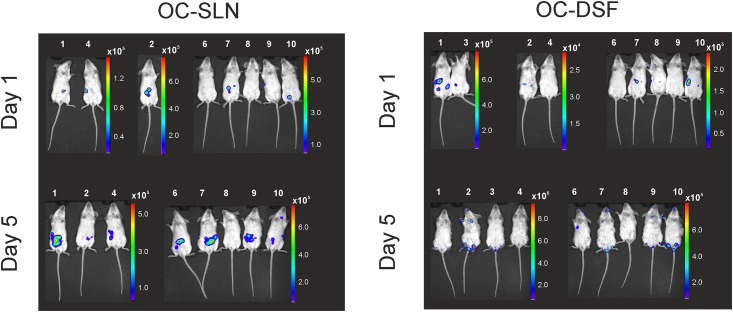
IVIS monitoring of the *Y*. *enterocolitica* O:3 OC gene cluster promoter activity in BALB/c mice infected i.g. with YeO3-Poc::*luxAB* reporter bacteria. The progress of the infection was monitored in DSF- and SLN-treated mice. The color bars beside each IVIS image indicate the intensity of radiance (p/sec/cm^2^/sr). Note that the scale range varies between the images due to different sensitivity settings used. The bioluminescence signals from live mice were monitored at 1 and 5 days post-infection. The images were acquired using a 5-min exposure with IVIS (Living Image^®^Software).

**Fig 5:**
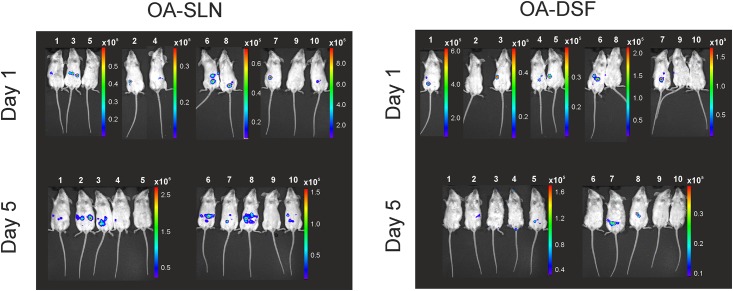
IVIS monitoring of the *Y*. *enterocolitica* O:3 O-ag gene cluster promoter activity in BALB/c mice infected i.g. with the YeO3-P_op1_::*luxAB* reporter bacteria. The progress of the infection was monitored in DSF- and SLN-treated mice. The color bars beside each IVIS image indicate the intensity of radiance (p/sec/cm^2^/sr). Note that the scale range varies between the images due to different sensitivity settings used. The bioluminescence signals from live mice were monitored at 1 and 5 days post-infection. The images were acquired using a 5-min exposure with IVIS (Living Image^®^Software).

On day one in both the OC-SLN and OA-SLN-infected mice the light emission was detected from 1–3 spots located at the intestinal region except in two mice where the spot close to the caecum (Fig 4, the OC-SLN mouse #10, Fig 5, the OA-SLN mouse #8). On day five, in both groups more intense but still spotwise light emission was detected from the intestinal region. Only in the OC-SLN mice #10 light emissions also occurred from the location close to the thyroid gland.

The picture on day one in the OC-DSF and OA-DSF groups was similar to that of the day one SLN-groups, i.e., light emission was detected from spots in the intestinal region. On day five, in contrast to the SLN-mice, the intestinal region light emission was almost absent, present only in four of the 20 mice (Figs 4 and 5). Instead, nine of the 20 mice had light emission from external parts of the body. Six of the DSF-group mice showed luminescence around the anus demonstrating that bacteria were secreted in the feces. All the nine DSF-mice had very likely developed diarrhea as they had soiled their external parts with the reporter bacteria-contaminated feces as the light emission was located in the front and hind paws, and around the snout.

**Fig 6:**
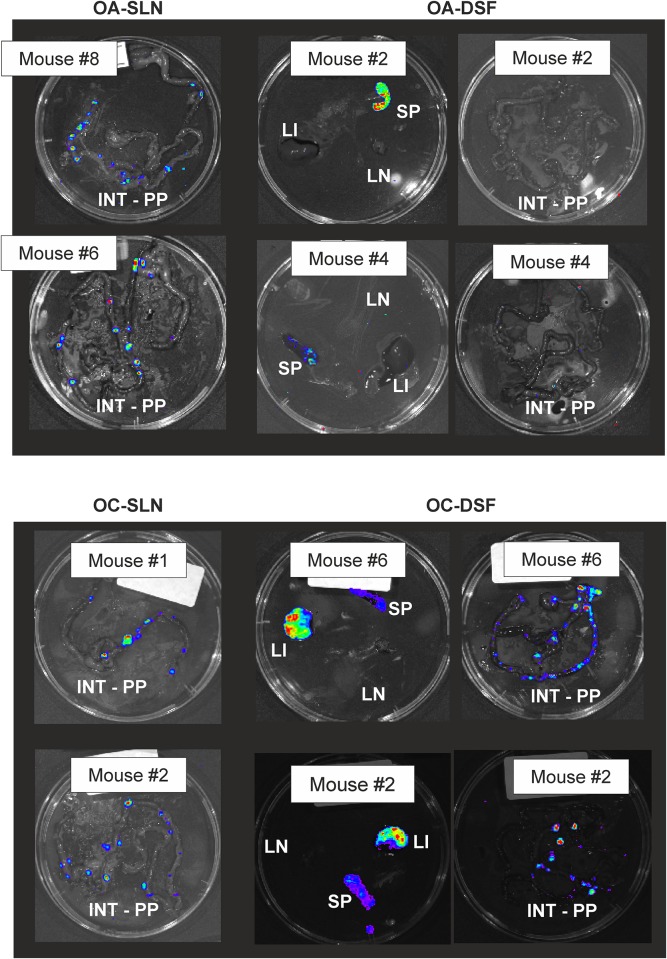
Representative images of light emission from excised mouse organs on day 5 post-infection captured by IVIS. The mouse numbering is the same as in Figs 4 and 5. Abbreviations: INT, intestine; SP, spleen; LN, mesenteric lymph node; PP, Peyer’s Patches; LI, Liver. The images were acquired using a 1-min exposure with IVIS (Living Image^®^Software).

On day 5 post-infection all the mice were sacrificed via cervical dislocation under anesthesia., the different tissues were recovered, light emission monitored by IVIS (Fig 6), and the bacterial loads in pooled tissues determined by colony counts (Table 4). In most mice clear light emission was detected from the Peyer’s patches of the excised intestines and especially in the DSF-group mice also from spleens and livers (Fig 6). The light emission from the spleen of the OA-DSF mouse #2 was strong enough to be detected also from the live mouse (Figs 5 and 6).

**Table 4:** Average bacterial loads in the pooled tissues of the IVIS experiment mice. Identical tissues of each mouse group were combined and homogenized for bacterial counts.

Mouse group	Tissue load (cfu/g)				
	INT	PP	MLN	SP	LI
**OC-SLN**	2.3×10^6^	3.0×10^5^	NG^a^	5.3×10^3^	NG
**OC-DSF**	5.0×10^5^	4.0×10^3^	2.7×10^3^	1.5×10^8^	3.8×10^5^
**OA-SLN**	1.4×10^5^	1.1×10^5^	NG	6.0×10^2^	NG
**OA-DSF**	5.0×10^4^	1.1×10^3^	NG	4.0×10^5^	1.6×10^6^

^a^NG, no growth. The detection limit was ca. 100 cfu/g. INT: intestine, SP: Spleen, MLN: Mesenteric Lymph Node, PPs: Peyer’s Patches, LI: Liver.

The major differences in the bacterial loads between the SLN and DSF groups were detected in spleen and liver. Clearly, DSF facilitated the bacterial colonization of these tissues. At the same time, no significant difference was detected in the colonization of the intestine and actually the bacterial loads in PPs were smaller in the DSF group (Table 4) reflecting well the strong light emission from the intestinal region of the OA-SLN and OC-SLN mice on day 5 (Figs 4 and 5).

IVIS detected strong light emission from excised spleens and livers of some mice (Fig 6). We did not determine the individual bacterial loads for the IVIS mice tissues, however, the average bacterial loads in the spleens and livers of the DSF-group mice were high (Table 4) explaining the strong signals from these organs.

## Discussion

In this work, we constructed a genome integrated and stable luciferase-based promoter reporter system for YeO3 that can be applied to direct *in vivo* imaging without need of administration of any substrate or antibiotics during infection. The *luxCDE*-carrying strain produces constitutively the luciferase substrate and will allow monitoring the activities of any promoter cloned upstream the promoterless *luxAB* genes. As a proof of principle, we introduced the LPS O-ag and OC gene cluster promoters into the *luxCDE*-strain and demonstrated that the reporter system is functional both *in vitro* and *in vivo*.

Various reporter systems have been developed to understand gene expression in different conditions [24, 41]. For instance, promoterless chloramphenicol acetyltransferase gene (*cat*) has been used to study promoter activity, however, while the *cat* reporter shows high sensitivity, the stability of the Cat protein is a disadvantage preventing research on rapid dynamic changes in expression [42]. The green fluorescent protein (GFP) and its derivatives are used as reporter although it is sometimes occluded by cellular autofluorescence [43]. Our chromosomally integrated *luxCDE* and *luxAB* genes allow self-sustainable production of the luciferase substrate and *in vivo* applications with continuous monitoring of gene expression. The merodiploid promoter construct appears to be stable *in vivo* as demonstrated by our preliminary mouse experiment where we infected mice either with wild type or reporter bacteria. The results demonstrated that the reporter bacteria (enumerated using Clm-plates) and the wild type bacteria colonized the mice to comparative levels, for example, the PPs were colonized equally to ca. 10^5^ cfu/gr (data not shown). If the reporter strains had been unstable, one would expect significantly less Clm-resistant colonies. As this was not the case, one can only conclude that the reporters are stable and as virulent as the wild type bacteria. The genome-integrated reporter plasmids are more stable than autonomously replicating reporter plasmids and, as in this work, can be used without feeding antibiotics to infected animals. While *luxCDABE*-carrying reporter strains have earlier been constructed for *Yersinia*, [25, 44–46] our system is the first one where the *luxAB* and the *luxCDE* genes have been separated.

The OC and O-ag gene cluster promoters are predicted to be most active *in vitro* during the exponential growth phase coinciding with strong need for new LPS molecules [9]. This was confirmed in this study. The promoters behaved differently at RT during the log phase where the Poc promoter activity was very low while that of Pop1 was higher (Fig 3). Previous reports using late log or stationary phase *Y*.*enterocolitica* bacteria demonstrated that O-Ag production is abundant in bacteria grown at RT while it is partially repressed at 37°C [47]. Regulation of many virulence factors in *Y*. *enterocolitica* is temperature depended. LPS is a significant virulence factor of *Y*. *enterocolitica* O:3 and both the OC and O-ag of LPS are required for full virulence [10, 14]. However, no experimental data of the regulation of the LPS biosynthesis of YeO3 *in vivo* is available. Our reporter constructs can be used to monitor tissue-specific expression of the OC and O-ag gene cluster promoters during *in vivo* conditions provided that the mice are killed to measure the luciferase activity and the bacterial loads of the infected organs. In order to use IVIS for that purpose, a dual-reporter system should be designed where a constitutively expressed reporter, for example GFP, would be used to normalize the luciferase activity to the number of bacterial cells colonizing the different tissues.

The reporter strains YeO3-Poc::*luxAB* and YeO3-Pop1::*luxAB* produced light during the *in vivo* experiments providing a proof of concept that the reporter system works *in vivo*. The Poc and Pop1 promoters were active throughout the intestinal region (Figs 4 and 5). The IVIS experiment showed that both promoter activities could be detected in the mice irrespective the DSF-treatment; however, DSF clearly influenced the course of infection. As a general observation, the DSF-group mice suffered from a more severe infection. This was also supported by the finding that light was detected from the excised liver and spleen tissues of OC-DSF and OA-DSF but never from the SLN-mice (Fig 6). This nicely reflected the fact that the bacterial loads in the DSF-treated mouse livers and spleens was much higher than in the SLN-treated mouse organs (Table 4).
